# Rationale and design of a randomized clinical trial evaluating the efficacy of mechanical neuroprotection in reducing the risk of silent brain infarcts associated with percutaneous left atrial appendage closure: study protocol for a LAAC-SBI trial

**DOI:** 10.1186/s13063-023-07766-3

**Published:** 2023-11-23

**Authors:** Witold Streb, Anetta Lasek-Bal, Katarzyna Mitręga, Jacek Kowalczyk, Tomasz Podolecki, Wiktoria Kowalska, Anna Olma, Agata Sobczyk, Zbigniew Kalarus

**Affiliations:** 1 Department of Cardiology, Congenital Heart Diseases and Electrotherapy, Faculty of Medical Sciences in Zabrze, Medical University of Silesia, Katowice, Poland; 2 Silesian Centre for Heart Diseases in Zabrze, Curie-Skłodowskiej 9, Zabrze, 41‒800 Poland; 3 Department of Neurology, Faculty of Health Sciences in Katowice, Medical University of Silesia, Katowice, Poland

**Keywords:** Silent brain infarcts, Atrial fibrillation, Neuroprotection, Therapeutic occlusion, Atrial appendage

## Abstract

**Background:**

Left atrial appendage closure (LAAC) procedures prevent cardioembolic stroke in patients with atrial fibrillation who have contraindications to oral anticoagulant medications. However, these procedures carry certain risks of peri-procedural complications. One such complication is silent brain infarcts (SBI), which can lead to cognitive impairment and mood disturbances. The implementation of mechanical neuroprotection systems during LAAC procedures may reduce the risk of SBI and associated cognitive and mood disorders.

**Methods:**

The LAAC-SBI trial is a prospective, multicenter, randomized, and double-blind interventional study. The study aims to enroll a total of 240 patients, with 120 patients allocated to each group. The study group will evaluate the use of the Sentinel CPS during LAAC, while the control group will undergo LAAC procedures without the Sentinel CPS. The primary endpoint of the study is the number of new SBIs or stroke foci detected by diffusion-weighted magnetic resonance imaging (DW MRI). Secondary endpoints include deterioration of cognitive function, development of dementia syndrome, and occurrence of depressive disorders. These endpoints will be assessed using questionnaire tools such as the Montreal Cognitive Assessment (MoCA), Trail Making Test (TMT), Controlled Oral Word Association Test (COWAT), and Hospital Anxiety and Depression Scale (HADS). The observational period for patients in the study is 2 years.

**Discussion:**

If the study demonstrates a favorable outcome with reduced incidence of SBI and improved cognitive and mood outcomes in patients receiving cerebral protection devices during LAAC, it will have significant implications for clinical management standards. This would support the use of neuroprotection devices not only for LAAC but also in procedures such as atrial fibrillation ablation or transcatheter mitral valve interventions, where the risk of embolic events and subsequent brain injury may also be present.

**Trial registration:**

ClinicalTrials.gov NCT05369195. Registration on 11.05.2022.

## Introduction

### Background and rationale {6a}

Atrial fibrillation (AF) is considered the most significant risk factor for stroke. A patient registry conducted across 47 countries revealed that approximately 4% of patients experienced a stroke within 1 year of being diagnosed with AF, and approximately 11% died [1]. Therefore, patients who obtain a score of ≥ 2 points on the CHA2DS2-VASc scale are classified as having Class I indications for stroke prophylaxis by the European Society of Cardiology (ESC). While oral anticoagulants remain the cornerstone of treatment, some patients may have contraindications to these medications. In such cases, transcatheter left atrial appendage closure (LAAC) serves as an alternative method for stroke prevention.

The effectiveness and safety of LAAC procedures have been established through extensive research involving randomized and observational trials [2–4]. Furthermore, it has been demonstrated that LAAC provides superior survival benefits compared to warfarin therapy [5]. However, it is important to acknowledge that the LAAC procedure carries certain risks, such as periprocedural stroke and microembolization of cerebral blood vessels, leading to SBI. The risk of periprocedural stroke is reported to be 0.17% [6]. Thus far, only limited data regarding the prevalence of LAAC-related SBI have been published. The incidence of SBI in these studies ranged from 4.8 to 52% [7–10]. Prospective and observational studies have provided evidence linking cognitive decline and the development of dementia to SBI [11]. A meta-analysis of 31 studies estimated the overall risk of dementia in AF patients with SBI at 1.48 [12].

#### Rationale

Evidence from randomized trials demonstrates that cerebral protection systems can effectively reduce the risk of complications such as stroke and SBI during transcatheter aortic valve implantation (TAVI). Devices like TriGuard, Sentinel Cerebral Protection System (CPS), and Embrella have been specifically developed for this purpose. The Sentinel CPS device has been evaluated in the randomized trials CLEAN-TAVI, MISTRAL-C, and Sentinel-H during TAVI procedures. The CLEAN-TAVI single-center study involved 100 patients randomly assigned in a 1:1 ratio to undergo TAVI procedures, either with or without neuroprotection. The study documented a statistically significant reduction in both the number and volume of new ischemic brain lesions observed on diffusion-weighted magnetic resonance imaging (DW-MRI) at 2 and 7 days post-intervention (39% and 51% reduction, respectively) in the protected regions and the entire brain (*p*-values ranging from < 0.001 to 0.02) among patients who received the Sentinel CPS compared to those receiving standard care without brain protection [13].

In the multicenter MISTRAL-C study, 65 patients were enrolled. Although there was a lower number of SBI foci and a reduced SBI volume (95 mm^3^ vs. 197 mm^3^) on DW-MRI in the group with the Sentinel CPS compared to the group without neuroprotection, the difference did not reach statistical significance. Furthermore, 27% of patients in the Sentinel CPS group had no new SBI occurrences, compared to only 13% in the control group. Additionally, multiple (> 10) SBI foci were only observed in the control group. Patients who used the Sentinel CPS device also exhibited less cognitive impairment on the Mini-Mental State Examination (4% vs. 27%; *p* = 0.017) [14].

The most extensive multicenter and prospective study, SENTINEL-H, involved 217 patients undergoing TAVI. Using the Sentinel CPS device was associated with minimal risk to patients, as indicated by the high success rate of device insertion and removal (94.4%) and only one case (0.4%) of access site vascular injury. Strokes occurred in 5.6% of patients treated with the Sentinel CPS, compared to 9.1% in the control group. Among patients who experienced a stroke, the size and number of DW-MRI lesions were reduced when the Sentinel CPS was employed. The study revealed a correlation between SBI volume and cognitive decline (*r* =  − 0.25; *p* = 0.002 for protected areas); however, the differences between the study and control groups were not statistically significant. The study’s authors attributed this finding to significantly reduced cognitive functions at the study’s baseline [15].

In the DEFLECT III study, which included 85 randomized patients, the TriGuard system was compared to no neuroprotection during TAVI. The use of the TriGuard system was associated with a higher rate of no new SBI occurrences (26.9% vs. 11.5%), a lower incidence of neurological deficits assessed by the National Institutes of Health Stroke Score (NIHSS) (3.1% vs. 15.4%), and better outcomes on the Montreal Cognitive Assessment (MoCA) test. Additionally, patients using the TriGuard system were twice as likely to experience cognitive function normalization in the MoCA test after 30 days (45.5% vs. 20%; RR 2.27 [95% CI 1.01, 5.1]) [16].

The Sentinel CPS has received approval to prevent embolism by capturing and removing embolic materials, such as clots or fragments, that may enter the cerebral vasculature during endovascular procedures. However, it should be noted that the embolic material encountered during TAVI procedures differs from the embolic material found in the left atrial appendage (LAA) in structure, quantity, and nature. No randomized trials have been conducted to specifically assess the impact of using neuroprotective devices during LAAC. Only isolated cases of utilizing such devices during LAAC procedures have been described. The largest reported sample includes six patients with a thrombus present in the LAA, in whom the implementation of brain protection devices prevented the occurrence of overt stroke. However, cognitive function assessments were not conducted before and after the procedure, and no brain imaging studies were performed [17].

### Objectives {7}

The objective of the present study was to investigate the occurrence of SBI and evaluate the impact on cognitive function and the prevalence of mood disorders in patients with AF who underwent LAAC utilizing the Amplatzer Amulet occluder, with or without the utilization of the Sentinel CPS. The study aimed to test the hypothesis that using the Sentinel CPS would mitigate the risk of SBI, cognitive decline, and depression in these patients.

### Trial design {8}

**Fig. 1:**
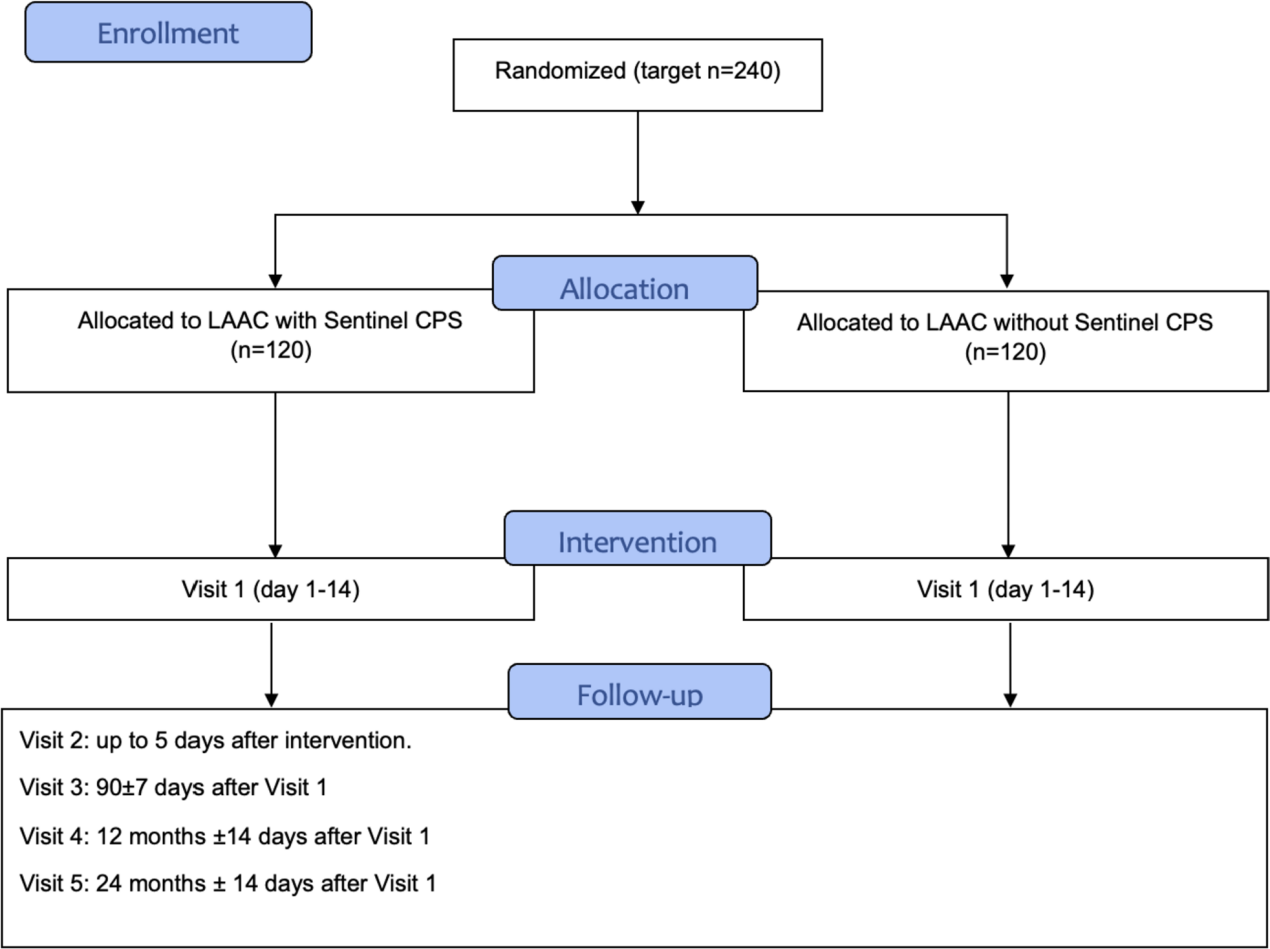
Flow diagram

The LAAC-SBI trial is designed as a prospective, multicentre, randomized, and double-blind interventional study. Its primary objective is to recruit a total of 240 patients, with an equal allocation of 120 patients to each group. The study group will examine the effectiveness of utilizing the Sentinel CPS during LAAC procedures, whereas the control group will undergo LAAC without using the Sentinel CPS (Fig. 1). The study was designed to demonstrate the superiority of an experimental procedure, i.e. the use of Sentinel CPS during LAAC, over a control intervention.

## Methods: participants, interventions, and outcomes

### Study setting {9}

This study is being started by an academic hospital. The study intends to involve eight centers located in Poland. You may find a current and complete list of these collaborating hospitals on ClinicalTrials.gov.

### Eligibility criteria {10}

The study will enroll patients who meet the following criteria:

- Aged ≥ 18 years
- Diagnosed with paroxysmal, persistent, or permanent AF
- ESC class I indications for preventing ischemic stroke, indicated by a CHA2DS2VASc score of ≥ 2
- Contraindications to anticoagulant use or a high bleeding risk based on a HAS-BLED score of at least 3 points

Patients meeting any of the following criteria will be excluded from the study:

- History of ischemic stroke
- History of central nervous system infections
- Neurological diseases such as Parkinson’s disease, Huntington’s disease, Creutzfeldt-Jakob disease, and Pick’s disease
- Severe mental disorders, including dementia of any etiology, schizophrenia, bipolar disorder, and depression
- Anatomical obstacles to the LAAC procedure or the insertion of a neuroprotection system
- Presence of mechanical heart prostheses

### Who will take informed consent? {26a}

Patients participating in the study must be familiar with the information presented in the informed consent form and give it in writing in the investigator's presence.

### Additional consent provisions for collection and use of participant data and biological specimens {26b}

The study does not provide for additional sample collection, which would require additional consent from study participants.

## Interventions

### Explanation for the choice of comparators {6b}

Patients enrolled in the study will undergo LAAC procedures conducted using the standard approach. However, only the study group will exclusively integrate the CPS Sentinel neuroprotection system during this procedure. The selection of this specific neuroprotection system stems from its approval for clinical use and its capability to safeguard approximately 95% of brain tissue from potential embolisms during intracardiac interventions.

Furthermore, the favorable outcomes observed when implementing the CPS Sentinel in patients undergoing TAVI procedures are compelling evidence of its high effectiveness. As a result, the researchers have chosen this system for investigation in the context of LAAC procedures to assess its potential benefits in reducing embolic events and enhancing patient outcomes.

### Intervention description {11a}

The LAAC procedure in the study group will involve the utilization of Amplatzer Amulet occluders along with the Sentinel CPS for cerebral circulation protection. Transcatheter LAAC interventions will be carried out under deep sedation or general anesthesia, using right femoral vein access.

The procedure encompasses several key steps to ensure accurate and safe placement of the Amplatzer Amulet occluder. Initially, a guide is positioned in the superior vena cava via the right femoral vein to establish a pathway for accessing the left atrium. Subsequently, a transeptal catheter is introduced and gradually withdrawn through the first and second slopes (foramen ovale) until access to the left atrium is achieved. This puncture process is meticulously monitored using fluoroscopy and transesophageal examination to ensure precise needle positioning and safety during atrial septum puncture.

Once left atrium access is established, a catheter is guided to the left atrial appendage (LAA) for angiography, providing a detailed assessment of LAA anatomy. Following this, the catheter is advanced to the left upper pulmonary vein and replaced with a specialized catheter designed for LAAC. The catheter is carefully positioned within the LAA at the appropriate depth, and a self-expanding Amplatzer Amulet occluder is inserted to close the LAA effectively. After successful occluder deployment, it is carefully pulled to verify proper positioning and assess the efficacy of LAA elimination. Finally, the guide catheter is removed, and a hemostatic suture is applied to the groin to ensure proper closure and minimize bleeding risk.

In the study group, before the LAAC procedure, a Sentinel CPS will be inserted to provide neuroprotection. Several steps are undertaken during the insertion of the Sentinel CPS. Firstly, arterial access is obtained through a radial artery puncture, and a pigtail catheter is inserted into the ascending aorta to perform aortography, assessing the aortic arch and cerebral arteries’ anatomy. The proximal filter of the Sentinel CPS is expanded in the brachiocephalic trunk, and the device is carefully rotated in the ascending aorta to position its tip in the left common carotid artery accurately. Subsequently, the distal filter of the Sentinel CPS is expanded, and fluoroscopy is used to ensure the correct positioning of both filters, thereby providing neuroprotection during the LAAC procedure.

After the successful implantation of the occluder in the LAA, the Sentinel CPS will be removed. The activated clotting time (ACT) must be maintained above 250 s during the procedure. Therefore, heparin will be administered. In the control group, heparin will be administered after the transeptal puncture. In the control group, the LAAC procedure will be performed in the same manner as in the study group but without installing the Sentinel CPS.

### Criteria for discontinuing or modifying allocated interventions {11b}

Analyses within the study will be carried out in accordance with the principle of intent-to-treat. Consequently, should a participant assigned to the study group not receive the Sentinel CPS intervention, they will still be retained within the study group. There will be no reassignment of participants between the study and control groups.

### Strategies to improve adherence to interventions {11c}

Given the interventional nature of this study, strategies for monitoring adherence pertain to the execution of follow-up visits and the associated procedures delineated for such instances. During follow-up visits, patients will be scheduled for subsequent appointments and receive timely telephonic reminders ahead of their impending appointments.

### Relevant concomitant care permitted or prohibited during the trial {11d}

Participation in the study does not influence the treatment of alternative medical conditions. Concerning prior therapeutic approaches, the patient enrolled in the study will not use oral anticoagulants after the procedure. Instead, they are advised to undergo dual antiplatelet therapy for 3 months post-LAAC intervention.

### Provisions for post-trial care {30}

The study incorporates an insurance policy designed to address any potential harm experienced by participants. After completing participation in the study, patients will no longer be obligated to undergo oral anticoagulant treatment as a component of stroke prevention in cases of atrial fibrillation. Instead, they will solely be recommended to take acetylsalicylic acid.

### Outcomes {12}

The study’s primary endpoint is the number of new SBIs or stroke foci in the DW MRI. In addition, the following secondary endpoints were defined:

- Volume of SBI and stroke foci in DW MRI
- Deterioration of cognitive functions assessment using standardized tests: The Montreal Cognitive Assessment (MoCA) test versions 8.1 and 8.3, Trail Making Test (TMT) parts A and B, Controlled Oral Word Association Test (COWAT)
- Development of dementia syndrome
- Occurrence of depressive disorders using Hospital Anxiety and Depression Scale (HADS)
- Presence of embolic material in the filters of the neuroprotection device
- Complications related to the use of the neuroprotection system during LAAC

### Participant timeline {13}

The participant timeline is shown in Table 1.

**Table 1:** Schedule of enrolment, interventions, and assessments

	**STUDY PERIOD**							
	**Enrolment**	**Allocation**	**Post-allocation**					**Close-out**
TIMEPOINT**	***Visit 0***	**Allocation**	***Visit 1***	***Intervention***	***Visit 2***	***Visit 3***	***Visit 4***	***Visit 5***
**ENROLMENT:**								
**Eligibility screen**	X							
**Informed consent**	X							
***Laboratory tests***	X							
***Transesophageal echocardiography***	X							
***Transthoracic echocardiography***	X							
***Carotid artery ultrasonography***	X							
***Electrocardiogram***	X							
***DW MRI***	X							
**Allocation**		X						
**INTERVENTIONS:**								
***LACC procedure***				X				
**ASSESSMENTS:**								
***DW MRI***					X			X
***Neurological assessment/NHSS***			X		X		X	X
***MoCA***			X		X	X	X	X
***TMT A&B***			X		X	X	X	X
***COWAT***			X		X		X	X
***HADS***			X				X	X
***EQ-5d-5L***			X				X	X
***Laboratory tests***			X		X	X	X	X
***Transesophageal echocardiography***			X			X	X	X
***Transthoracic echocardiography***			X				X	X
***Carotid artery ultrasonography***								X

### Sample size {14}

Based on the assumptions that cerebral protection during LAAC would result in a favorable response in 85% of patients and that the absence of new SBI on DW MRI in the control group would affect 70% of patients, the minimum group size was estimated to be 118 individuals per group. This estimation ensured 80% power to detect the anticipated difference at a significance level of 5%. Considering a withdrawal or invalidation ratio of 2%, a total of 120 individuals will be enrolled in each group, resulting in a sample size of 240 patients for the study. This sample size is necessary to provide sufficient statistical power and enable meaningful comparisons between the study and control groups.

### Recruitment {15}

Medical centers known to conduct a substantial volume of LAAC procedures were invited to participate in the study. Among individuals referred to these centers, patients eligible to participate in the study will be selected based on the inclusion and exclusion criteria.

## Assignment of interventions: allocation

### Sequence generation {16a}

Block randomization will be employed to uphold the proportional allocation of subjects across the two groups. The blocks will consist of four subjects each. Randomization will be centrally executed to mitigate the foreseeability of the forthcoming treatment modality for successive patients. This will be accomplished by utilizing existing computer algorithms facilitated by the Interactive Web Response System.

### Concealment mechanism {16b}

The Sentinel CPS is introduced via a radial artery access and is preceded by aortography. To minimize subjectivity during patient evaluations of at follow-up visits, individuals within the control group will similarly undergo arterial puncture and aortography, mimicking the setup process for the Sentinel CPS. Furthermore, the neurologist conducting the neurological examination, utilizing the NIHSS score and cognitive functionality assessments, will be kept unaware of the patient’s allocation to either study arm. They will also not have access to the eCRF to prevent any knowledge about the patient’s assignment to the study or control group. This approach, known as “investigator blinding,” aims to ensure impartial evaluation.

### Implementation {16c}

Unblinded researchers will enroll patients at each participating medical center based on predetermined inclusion and exclusion criteria. Additionally, these researchers will carry out patient randomization utilizing a central computer system. External monitors to the study sponsor will oversee the accuracy of patient inclusion and adherence to the randomized assignments. These monitors are affiliated with the BioStat Sp. z o.o., a Clinical Research Organization (CRO) specializing in comprehensive research services. The study Sponsor has established a contractual agreement with this CRO for these monitoring purposes.

## Assignment of interventions: blinding

### Who will be blinded {17a}

Both the patient undergoing assessment and the neurologist conducting the physical examination and cognitive tests will be kept uninformed regarding the utilization of neuroprotection with the Sentinel CPS during the LAAC procedure. Furthermore, DWI MRI images will be evaluated at a central laboratory separate from the centers participating in the study. The radiologist responsible for analyzing the DWI MRI images will also remain unaware whether the patient was assigned to the study or control groups. This approach ensures an unbiased and impartial assessment of outcomes.

### Procedure for unblinding if needed {17b}

Given that the Sentinel CPS is employed to prevent perioperative stroke, details concerning its utilization remain independent of managing potential perioperative complications or the emergence of new medical conditions during the follow-up period. Consequently, it is anticipated that no unblinding procedures will be implemented throughout the study.

## Data collection and management

### Plans for assessment and collection of outcomes {18a}

For the study, researchers must possess a valid Good Clinical Practice certificate. To acquire robust data regarding the efficacy of neuroprotection during LAAC procedures and to evaluate the clinical advantages of employing the Sentinel CPS, both imaging data—specifically brain DWI MRI—and assessments of cognitive functions and mood disorders will be collected. To ensure the accuracy of DWI MRI data, these images will undergo analysis at a central laboratory. Additionally, detailed instructions regarding the DWI MRI protocol will be furnished to participating centers.

Conversely, the evaluation of cognitive functions and mood disorders will involve the utilization of validated questionnaires. The use of these questionnaires is authorized by entities holding the respective copyrights. Researchers tasked with administering the Montreal Cognitive Assessment (MoCA) test must hold certification for conducting and interpreting the test. This certification is granted after completing appropriate training, available at mocacognition.com.

### Plans to promote participant retention and complete follow-up {18b}

To ensure participants’ continued engagement in the study, a telephone reminder will be implemented before the upcoming follow-up visit. If a participant becomes unable to attend follow-up visits due to health-related or other reasons (excluding withdrawal of informed consent), the researcher will make diligent attempts to document the observation comprehensively. This will involve initiating telephone correspondence with the participant, contacting their family, or liaising with their healthcare provider or relevant institution/authority. These measures are intended to facilitate remote interviews and accurately assess the participant’s condition.

### Data management {19}

A data management plan version 1.0 has been prepared, the purpose of which is to present the procedures and control mechanisms necessary to ensure the protection, authenticity, confidentiality, completeness, and integrity of data collected from study participants, as well as the mechanisms necessary to ensure that the clinical database created as part of the study will be complete, cleaned and usable for the Sponsor and statisticians to analyze the collected data and prepare the clinical trial report. This document has been prepared in accordance with the guidelines of “Good Clinical Data Management Practices” and the regulations of 21 CFR Part 11. This document will be updated throughout the study to reflect changes to the electronic Case Report Form (eCRF) and comments from Monitors, Sponsor, Investigators, and Coordinators. Clinical data for the LAAC-SBI study is collected through the eCRF.bizTM system, an Electronic Data Capture platform developed by BioStat. This system is fully validated and serves as the repository for study-related information. The eCRF.bizTM system operates on a server running the GNU/Linux Debian 11 operating system. It utilizes PHP version 7.2.34, MySQL database version 8.0.29–2, and Apache web server version 2.4.54. Certain functionalities linked to reporting and encoding medical data involve using the R statistical software (version 3.3 or later) and the Python programming language (version 3.5). To ensure security, the system has been situated on a secure server hosted at the data center of OVH, a prominent European service provider known for its reliable infrastructure.

### Confidentiality {27}

All patient data entered into the eCRF are anonymous and identifiable by a unique patient number generated when creating the patient card. The research team is responsible for keeping information about the patient’s assigned number in the records kept at the site.

### Plans for collection, laboratory evaluation, and storage of biological specimens for genetic or molecular analysis in this trial/future use {33}

The current study does not plan to obtain biological material for genetic and molecular research.

## Statistical methods

### Statistical methods for primary and secondary outcomes {20a}

The primary analysis of all endpoints will be conducted using an intention-to-treat approach, wherein participants will be analyzed according to their initial randomization assignment, irrespective of the actual treatment received. Quantitative variables will be presented as either mean or median values, while qualitative variables will be reported as counts (with percentages). To assess the distribution of quantitative variables, the skewness index will be employed. Between-group comparisons will be carried out using independent *t*-tests and ANCOVA for quantitative variables. A chi-square test will be used to compare distributions between the two groups for qualitative variables.

### Interim analyses {21b}

The monitoring committee will act in an advisory role to ensure safety by reviewing the cumulative data from the clinical trial at specified intervals. Its tasks also include monitoring the clinical trial’s current validity and scientific value. The monitoring committee may recommend modifying or terminating a clinical trial based on any perceived safety concerns, regardless of statistical significance. The sponsor is authorized to stop the study at any time if the safety of the participants is compromised. The test may end prematurely, especially if:

- Serious adverse events outweigh the previously positive balance of benefits and risks,
- Adverse events occur with such intensity and/or frequency that the proposed treatment regimen can no longer be continued.

The sponsor is responsible for reporting the discontinuation of the trial to the Bioethics Committee, giving the reason for discontinuing the trial, and informing in writing about the potential risk to the health of clinical trial participants or other persons.

### Methods for additional analyses (e.g., subgroup analyses) {20b}

No subgroup analysis is planned.

### Methods in analysis to handle protocol non-adherence and any statistical methods to handle missing data {20c}

Instances where the procedure is not conducted per protocol, must be documented along with the underlying reasons. The analysis will still include data regarding the study participants who did not undergo the procedure aligned with their randomized group. The CRO and the Sponsor will regularly review the Protocol Deviation Reports. Should repeated deviations from the study protocol occur, the Sponsor reserves the authority to suspend recruitment at a particular center based on their judgment and decision-making.

Multiple imputations will be employed to address these gaps when data is missing for the independent (explanatory) variables. This method estimates the missing values based on the observed data, ensuring a more complete dataset for analysis. Conversely, for the dependent (outcome) variable, cases with missing data will be removed (using listwise/case deletion). The analysis will then be conducted solely on the remaining complete cases, utilizing the observed values of the outcome variable. This approach aims to maintain the integrity of the analysis while accounting for missing data in a systematic manner.

### Plans to give access to the full protocol, participant-level data and statistical code {31c}

The protocol may be made available by the principal investigator upon reasonable request.

## Oversight and monitoring

### Composition of the coordinating center and trial steering committee {5d}

The study's sponsor is the Silesian Centre for Diseases in Zabrze, which also serves as the coordinating center for the study. Managing the Clinical Research Organization tasks has been delegated to an external organization named BioStat sp z o.o. The Silesian Centre for Heart Diseases in Zabrze is responsible for establishing contracts with other participating centers and providing the necessary equipment and materials for conducting the study.

Furthermore, a Steering Committee has been instituted to oversee the clinical trial’s scientific and operational aspects. This committee convenes regularly to supervise participant recruitment, overall data collection, and any instances of site-specific non-compliance with the study protocol. It also evaluates and follows up on recommendations by the monitoring committee, addresses operational concerns that might arise warranting modifications to the study protocol or corrective actions, and establishes a policy for publishing findings from the clinical trial’s collected data.

### Composition of the data monitoring committee, its role and reporting structure {21a}

Team of Data Managers BioStat Sp. z o. o. is responsible for data management and monitoring. The eCRF.bizTM system has many tools to monitor the progress and status of the study in real-time. In addition, the clinical database of the LAAC-SBI study has been equipped with validators and transition rules. To ensure adequate data quality control, the Data Managers team of BioStat Sp. z o. o. will apply additional data verification procedures on the database snapshot exported from eCRF.bizTM to personal computers. To increase the accuracy and repeatability of verification, Data Managers will develop a series of scripts in the R statistical program and use MS Excel. The quality control of the clinical database dumps will take place at the beginning of the study after the inclusion of each subsequent ten patients (however, at intervals not longer than 1 month). Once all patients are included, the verification frequency will increase to at least one check per week.

### Adverse event reporting and harms {22}

Any Adverse Events (AEs) or Adverse Device Events (ADEs) that meet the criteria for severity must be promptly reported to the Sponsor within 24 h of their occurrence or as soon as awareness of their occurrence is established. The same procedure applies to instances of Product Defects that could potentially lead to Serious Adverse Events (SAEs) if appropriate action had not been taken, interventions were not carried out, or circumstances were less favorable.

On an annual basis throughout the study duration, the Principal Investigator will furnish the Ethics Committee that issued the study’s approval with a comprehensive list encompassing all suspected serious adverse events that transpired within that specific year. Furthermore, an annual report about patient safety will also be submitted. Information related to all SAEs will be incorporated into the finalized Clinical Study Report.

### Frequency and plans for auditing trial conduct {23}

The Clinical Research Associate (CRA) employed by BioStat Sp. z o. o. is tasked with overseeing the following study-related visits:

- Initiation Visit: This visit occurs prior to center activation to verify its readiness to initiate the study. The monitor assesses facility equipment, explains relevant regulations and protocol requirements, and conducts any necessary staff training.
- Monitoring visits: These visits are conducted to protect study participants’ rights, adherence to the protocol and applicable regulations (including Good Clinical Practice), accurate collection and reporting of safety data, and study endpoints.
- Closing Visit: The closing visit is conducted to ascertain the completeness of study data and documentation, confirming that all required audit-related procedures have been executed.

The frequency of monitoring visits to each center is contingent on the pace of patient recruitment and the quality of work. The study accommodates up to 64 monitoring visits. Around 60 patients will be included in the Source Data Verification pool, enabling the verification of approximately 25% of the data. Following each monitoring visit, the CRA will compile a visit report and send a summary letter detailing the visit’s outcomes to the center.

### Plans for communicating important protocol amendments to relevant parties (e.g. trial participants, ethical committees) {25}

To ensure the flow of all relevant and up-to-date information regarding the audit on an ongoing basis, the implementation of updated documents describing the procedures, compliance with deadlines, and conducting training, detailed requirements regarding the method and frequency of communication were set out in the Project Management Plan. The first contact person for the Sponsor is the Project Manager (PM) at Biostat Sp Z o.o. The PM also acts as the initial point of contact for the monitoring team. In turn, the PM provides the Sponsor and the monitoring team with all information requiring their attention on an ongoing basis. In urgent situations, especially those concerning safety in the study, the monitoring team/Sponsor will also send their notification by e-mail to the PM and the entire team.

The first point of contact for the center is the CRA. The CRA should contact the center to conduct necessary training and discuss current topics such as available study updates, deadlines, recruitment, eCRF completion and response to data input queries, center supplies, and other open issues. Any protocol amendments will be reported to the Ethics Committee and registered to ClinicalTrials.gov.

### Dissemination plans {31a}

The study results will be announced through peer-reviewed publications and conference reports.

## Discussion

LAAC procedures have emerged as a minimally invasive alternative for stroke prevention in patients with non-valvular AF, offering an alternative to long-term anticoagulant use and its associated bleeding risks. Randomized trials have demonstrated that LAAC is non-inferior to warfarin or new oral anticoagulants in terms of efficacy for stroke prevention. However, these procedures are not without risks, particularly during the peri-procedural period. While life-threatening complications such as stroke, cardiac tamponade, and device embolization have been reported, the incidence of SBI and their potential impact on cognitive and mood disorders have not been extensively studied in large trials. It is crucial to fully understand the potential harms associated with LAAC procedures to guide appropriate pharmacotherapy and the use of neuroprotection devices.

Moreover, the issue of SBI and the need for neuroprotection devices extends beyond LAAC and can be relevant to other endocardial procedures like AF ablation or percutaneous mitral valve procedures. The presence of a thrombus in LAA is a contraindication for these procedures. However, in clinical practice, the situation can be more complex, as small micro embolic foci may arise from damaged tissue during embolization of the cerebral circulation. Additionally, decreased left atrial flows can result in echocardiographic findings of self-contrasting blood or sludge, indicating a higher risk of SBI. Therefore, considering the application of mechanical neuroprotection to a broader patient population undergoing these procedures may be reasonable. Importantly, data from the literature suggest that neuroprotection devices offer high safety in routine clinical practice.

In summary, while LAAC procedures have demonstrated efficacy in stroke prevention, the potential risks, including SBI and its impact on cognitive and mood disorders, must be thoroughly evaluated. The potential beneficial effect of cerebral protection devices in reducing the risk of SBI and the subsequent cognitive impairment and mood disorders associated with LAAC procedures would have significant implications for clinical management standards. The findings of this study can potentially shape guidelines and recommendations for the use of cerebral protection devices during LAAC and other left atrial interventions, such as AF ablation. If the study demonstrates a favorable outcome with reduced SBI incidence and improved cognitive and mood outcomes in patients receiving cerebral protection devices during LAAC, it suggests that similar benefits could be extrapolated to other left atrial interventions. This would support the use of neuroprotection devices in procedures like AF ablation, where the risk of embolic events and subsequent brain injury may also be present. However, it is important to await the current study’s results to evaluate the impact and benefits of cerebral protection devices on SBI and related outcomes in patients undergoing LAAC.

## Trial status

According to version 1 of the protocol, the recruitment of participants for the study started on 26 May 2023. Patient enrollment for the study is anticipated to conclude by September 2025.

## Data Availability

Upon request, the Principal Investigator will provide public access to the final trial dataset for research purposes, contingent on assessment by the steering committee.

## Abbreviations

ADE: Adverse Device Event
AE: Adverse Event
AF: Atrial fibrillation
CPS: Cerebral Protection System
COWAT: Controlled Oral Word Association Test
CRA: Clinical Research Associate
CRO: Clinical Research Organization
eCRF: Electronic Case Report Form
ESC: European Society of Cardiology
HADS: Hospital Anxiety and Depression Scale
LAA: Left atrial appendage
LAAC: Left atrial appendage closure
MIS: Memory Index
MoCA: Montreal Cognitive Assessment
PM: Project Manager
SAE: Serious Adverse Event
SBI: Silent brain infarcts
TAVI: Transcatheter aortic valve implantation
TMT: Trail Making Test
